# A first evaluation of the usefulness of Kudzu starch in cultural heritage restoration

**DOI:** 10.1038/s41598-020-72643-x

**Published:** 2020-09-24

**Authors:** E. Lama, M. Veneranda, N. Prieto-Taboada, F. L. Hernando, M. D. Rodríguez Laso, J. M. Madariaga

**Affiliations:** 1 Department of Painting, Faculty of Fine Arts, University of the Basque Country UPV/EHU, P.O. Box 644, 48080 Bilbao, Basque Country Spain; 2 Department of Analytical Chemistry, Faculty of Science and Technology, University of the Basque Country UPV/EHU, P.O. Box 644, 48080 Bilbao, Basque Country Spain; 3 Department of Applied Chemistry, Faculty of Chemistry, University of the Basque Country (UPV/EHU), Pº Manuel Lardizabal, 3, 20018 Donostia, Spain; 4 Department of Immunology, Microbiology and Parasitology, Faculty of Science and Technology, University of the Basque Country UPV/EHU, P.O. Box 644, 48080 Bilbao, Spain

**Keywords:** Biochemistry, Chemistry, Materials science

## Abstract

In recent times, the use of natural and harmless products for the environment and restorer is taking place in the field of Cultural Heritage restoration. In this sense, wheat, rice and corn starches as adhesives, have suitable characteristics without toxicity risks. A new starch in this field, is the Kudzu, an almost pure compound (99.5% starch) that is processed by a natural way from a plant called *Pueraria lobata*. This is a preliminary study of the potential use of Kudzu starch for the restoration of Cultural Heritage, focusing, firstly, in its capacity as adhesive through a comparative evaluation with common starches. The accelerated aging process carried out proved that Kudzu ensures optimal chromatic behaviour. On the other hand, the main problem in starch paste is the biological colonization. The daidzein, a natural antimicrobial compound implicit in Kudzu starch, confirmed the resistance to microorganism in this preliminary approach. The evaluation of the adhesive capacity, and the reversibility of the starches, suggest that Kudzu starch is a valid adhesive in the field of paper restoration. Thus, the potential of this starch in the conservation of Cultural Heritage is evidenced and its use as cleaner, resistance to biological colonization and consolidant is promising.

## Introduction

The restoration and conservation field has evolved towards a work methodology more harmonious with the materials composing the artworks, as well as, their future conservation and the environment^1^. New advances in this field of research recently led to a critical improvement in the formulation of conservation products used regularly in museums, ateliers and restoration´s schools. In this sense, new materials and new procedures in art conservation have developed and enhanced thanks to a close collaboration with the experimental sciences^2^.

The employment of products harmful to the artwork, the environment and the restorers was relatively common until a couple of decades ago. Overall, these products, through purifications, mixtures and synthetizations, achieve optimal short-term results. However, in the long term, they mostly produce irreversible damages to the artworks, to the restorers and finally, to the environment due to an incorrect management of the residuals. Thanks to the conjugation of these two elements: science and restoration, it has been possible to study the behaviour of natural materials (in the line of green chemistry), which have been used for centuries in the crafting of artistic works and their subsequent restoration till the arrival of synthetic products some decades ago^1^.

One of the most used products within this natural product, is starch. This is already present since ancient times, in large processes, such as: tear repair in canvas restoration^3^, skin adhesion^4^ and is currently being investigated as an inclusion material in restoration lime mortars^5^. In this regard the used starches are those of wheat, rice and corn as their qualities and characteristics are known. Even thought, last two are commonly used in restoration works^6^.

In this sense, *Pueraria Lobata* where Kudzu starch is extracted, is a climbing plant related to the pea plant and endemic to Japan, and nowadays, it is expanded by many countries. It is catalogued by the International Union for Conservation of Nature (IUCN) as one of the one hundred most damaging invasive species in the world^7,8^. However, in Japan its root is extracted through a natural and ecological process to be used in traditional medicine and food industry. It is washed, dried and processed in a sustainable way, achieving a very good percentage of starch in the final product (around 99.5%) with a significantly high gelatinization temperature between 65 °C and 80 °C)^9,10^. The use of this root is, as has been previously mentioned, principally in traditional medicine because the presence of daidzein^11^, a flavonoid from the group of isoflavones that acts as a natural antimicrobial, is a recognized antifungal which is not present in other common starches. This compound can be found in different proportions, varying from 1 to 2% depending of the type of the Kudzu starch^12–14^.

Natural adhesives are frequently use in restoration of cellulose supports especially starch, either from wheat, rice, or corn depending on the material to bond^15^. Little by little, this material from a natural source is recovered again in European and American restoration field^16^, previously and nowadays this operation was carried out with products of synthetic or semi-synthetic origin derived from cellulose^17^, methylcellulose (thylosa 300)^18^, sodium carboxymethyl-cellulose^18^ or hydroxypropyl-cellulose (Klucel)^19^. Methylcellulose and sodium carboxymethyl are not good at adhering to smooth-surface papers or matboard^18^, but it has good aging and does not tend to yellow over time. The hydroxypropyl-cellulose is a stable product with a lower adhesive capacity than methylcellulose and sodium carboxymethyl-cellulose. It has a particular advantage, which can be used in polar organic solvents, avoiding water in certain paper supports^19^.

Taking into account that starches can be easily colonized by microorganisms, the natural presence of daidzein make Kudzu a promising product to be used as biocide or resistant, especially in paper restoration.

Despite these characteristics, Kudzu has never been used in the field of restoration and conservation of artworks. In this light, the main goal of this initial stage of study is to test the feasibility of Kudzu (*Pueraria Lobata*) in the conservation of Cultural Heritage used, firstly, as a novel adhesive product for paper restoration. Its performance will be compared with the most used starches in this field (rice and wheat, followed by one less commonly used corn, which is not as popular as the previous ones^6^) evaluating their capacities.

## Results and discussions

### Elemental and molecular characterization of the starches

Starch is not really a polysaccharide, but the mixture of two, amylose (a linear polymer of a-d-glucose units linked by a-1,4 glycosidic linkages) and amylopectin (a branched polymer of a-d-glucose units linked by a-1,4 and a-1,6 glycosidic linkages), representing approximately 98–99% of the dry weight. Starches contain between 20 and 30% amylose, although there are exceptions and each type of starch has its own proportions. These polymers have the same basic structure but differ in their length and degree of branching, which ultimately affects their physicochemical properties^10^.

**Figure 1:**
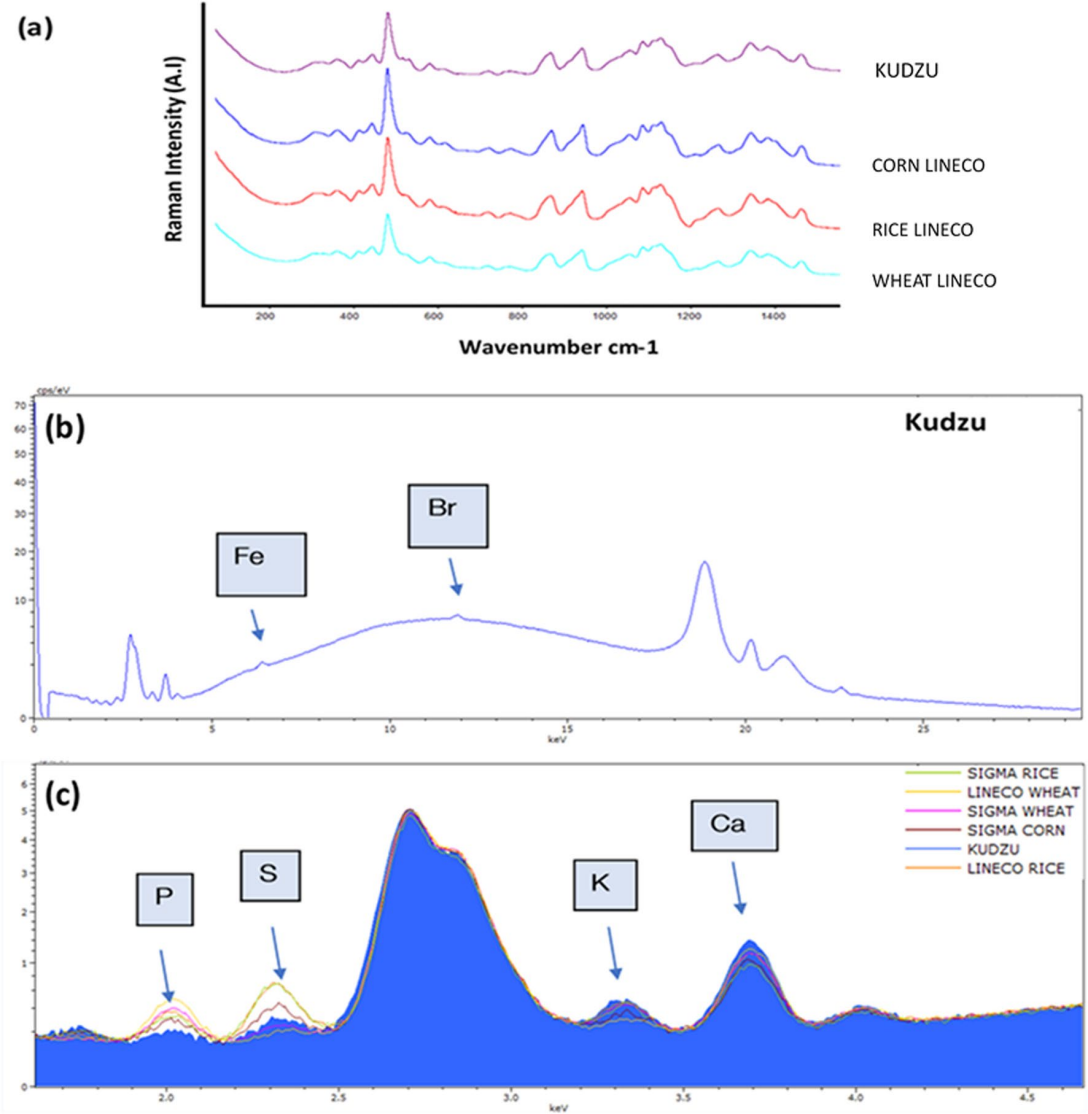
(**a**) Raman spectroscopy spectra of the commercial starches in Restoration field. (**b**)The Kudzu spectra revealed a few concentrations of Fe and Br. (**c**) Overlay spectra of starches. It observed the difference in the traces of some elements.

The Raman spectroscopic analyses gave an identical result in the four analysed starches, including Kudzu. Indeed, as can be seen in some examples of the starches analyzed, Fig. 1, the main Raman band appears at 478 cm^−1^, followed by the most significant secondary peaks at 1,125 cm^−1^ and 442 cm^−1^. Sigma starches also presented these identical peaks in their Raman spectrum. Taking into account the detection limit of the Raman spectrometer, any other compounds were observed indicating the purity of the used samples of the different starches.

In the case of elemental analysis, very similar EDXRF spectra were collected. However, it must be noted a slightly change in the concentration of trace elements on each starch.

Bromine, calcium, iron and sulphur were observed in all starches, being their spectroscopic signals higher in the Kudzu starch. The Lineco wheat spectrum contained less concentration of calcium than Kudzu but even so, greater than the others. Moreover, this starch given a higher phosphorus concentration than the rest, unlike the Kudzu that fallen short of this element. In all cases, the concentrations were at the level of traces.

### Colorimetric analysis and UV induced fluorescence after aging

The mean results obtained in the ageing process after the colorimetric analysis are collected in Table 1. As can be seen in the table, in all cases the covered samples and the control paper provided minimal colour variation (ΔE) which indicates the effect of UV light. Regarding covered samples, very small variations regarding the control sample were observed. Using the ASTM 4303-3 standard that classifies the total variation of colour in 5 categories, from the imperceptible to the human eye to the most remarkable variation (Table 2), the variation of all covered samples was imperceptible (Category 1). However, differences can be observed in the uncovered samples being all the changes perceptible and categorized as 2, and the best results were obtained by Kudzu starch, the one providing the closest results to the control paper. The change in the colour had a clear tendency to the yellow (high values of b parameter) one of the most important problems in the use of these kind of materials^20^ being Kudzu the less affected one.

**Table 1:** Mean results obtained after the Colorimetric analysis.

	Uncovered samples				Covered samples			
	a	b	L	**∆**E	a	b	L	∆E
Wheat	− 1.99	6.90	− 0.01	7.18	− 0.07	0.02	1.25	1.25
Rice	− 2.09	7.34	0.05	7.63	− 0.01	− 0.26	1.20	1.23
Kudzu	− 1.92	6.14	0.49	6.46	0.05	− 0.58	1.40	1.52
Control	− 1.82	6.00	0.06	6.30	0.26	− 1.09	1.00	1.50

The values are the variation regarding the initial and final measurements. ΔE (total colour variation) = [a2 + b2 + L2]1/2. The uncertainty associated to these measurements is around 5%

**Table 2:** Classification of the total colour variations depending on the values of ∆E.

Category 1	E*ab ≤ 4
Category 2	4 < E*ab ≤ 8
Category 3	8 < E*ab ≤ 16
Category 4	16 < E*ab ≤ 24
Category 5	E*ab ≥ 24

Regarding the results obtained during the long-term time period, it was not observed differences between the behaviour of the starches or the control paper, observing the major colour change in the first 150 h.

**Figure 2:**
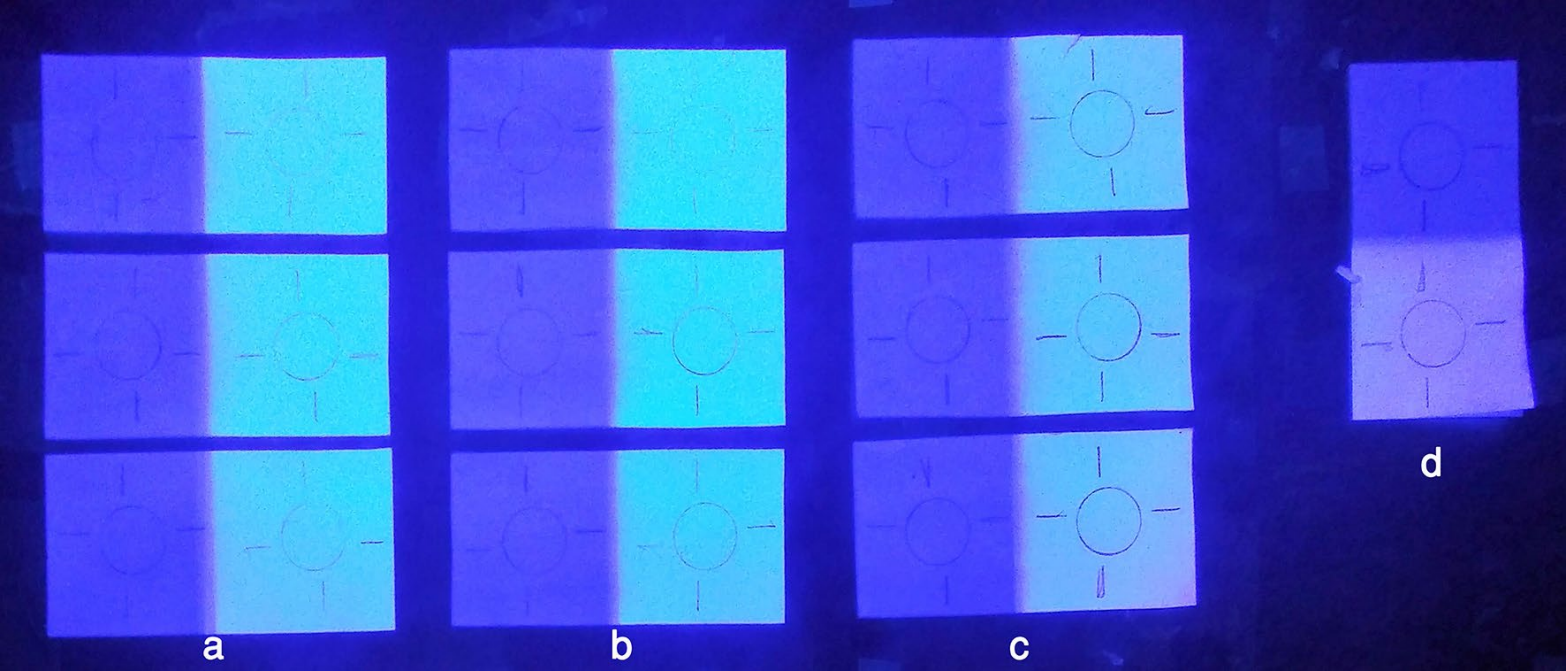
Image of the samples after the aging process with direct UV light of (**a**) Wheat, (**b**) Rice, (**c**) Kudzu and (**d**) Paper in which is possible to observe the effect of the radiation to the materials being Kudzu the less affected starch.

The image represented in Fig. 2, was collected under UV–Vis light and clearly displays the effect of the UV radiation in the starches and the paper. In fact, the starch and paper not exposed to the radiation remains dark, but the exposed starch and paper appeared with fluorescence indicating the occurrence of degradation processes. As can be seen Fig. 2c, the Kudzu is the less affected starch.

### Adhesion test

It is well known that all the starches have a certain adhesive power due to their hydrogen bonds, however not all the starches have exactly the same power and it must be therefore evaluated^20^. In addition, Sigma-Aldrich rice and wheat starches have greater adhesiveness than the rest with results of 2,123 × 10^–4^ N/mm and 1938 × 10^–4^ N/mm, respectively. Similar values of rice starch were obtained for Kudzu and Lineco rice starches with 1757 × 10^–4^ N/mm and 1871 × 10^–4^ N/mm respectively. Lastly, Lineco wheat starch and Sigma-Aldrich corn starch obtained the lowest results in adhesive strength (Table 3). Thus, Kudzu starch demonstrates a considerable strength of adhesion, being similar or even higher than starches commercialized as paper restoration products. The best results were obtained from Sigma-Aldrich compounds, which are not commercialized for restoration purposes. This characteristic is probably due to the higher purity of the starch. For this reason, the results obtained by Kudzu starch were very positive being the 94% of the adhesion capacity of Lineco rice starch and 88% of the Sigma-Aldrich rice starch.

**Table 3:** Average values of the adhesion forces (N/mm).

Adhesion	Starch	N/mm
–	Corn Sigma-Aldrich	1,449 × 10^–4^
	Wheat Lineco	1,604 × 10^–4^
	Kudzu	1,757 × 10^–4^
	Rice Lineco	1,871 × 10^–4^
	Wheat Sigma-Aldrich	1,938 × 10^–4^
+	Rice Sigma-Aldrich	2,123 × 10^–4^

### Study of microbiological growth

The proliferation area of the microorganisms in all the starches were from 40% onwards, reaching 70% of the culture dish (Table 4). The exception was Kudzu starch with only 10% of colonized area.

**Table 4:** Results of the growth of microorganisms in starch paste.

	Number of colonies	Number of different colony types	Size of the colonies diameter (mm)	Growth in petri dish (%)
Sigma-Aldrich rice	19 CFU	4–5	10–20	60–70
Sigma-Aldrich corn	31 CFU	3–5	5–15	55–60
Sigma-Aldrich wheat	27 CFU	3–5	3–10	40
Lineco rice	31 CFU	3–5	2–10	50
Lineco wheat	33 CFU	3–4	2–10	50
Kudzu	12 CFU	2–3	2–5	10

*CFU Colony forming units.

The number of initial and separate individuals.

**Figure 3:**
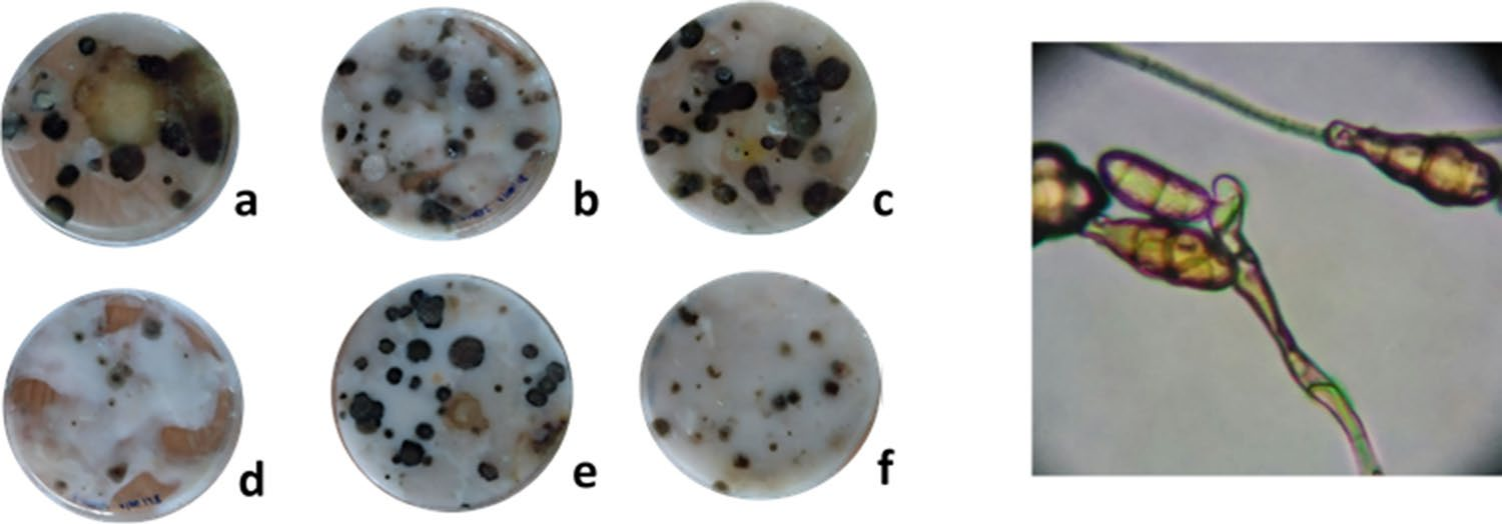
Image of the growth of microorganisms in the different starches. (**a**) Sigma-Aldrich rice, (**b**) Lineco wheat, (**c**) Lineco rice, (**d**) Kudzu, (**e**) Sigma-Aldrich corn and (**f**) Sigma-Aldrich wheat. In the right, image of Alternaria fungus which was found in all the samples.

In the commercial starches used in restoration (Lineco) appeared between 3 and 5 different types of colonies, on the contrary, in the Kudzu dish between 2 and 3 types of colonies were found (Fig. 3), occupying a surface markedly smaller. The most repeated microorganisms analysed were *Alternaria spp* (Fig. 3) and *Aspergillus spp* especially in wheat starches (Sigma-Aldrich and Lineco).

Regarding the number of colonies, the Kudzu registered 12 colony forming units (CFU) followed by the rice starch Sigma with 19 CFU. The most damaged starches against the biological attack were Lineco brand.

In all cultivated starches (except for Kudzu) the microbiological growth started at the fourth day. In contrast, Kudzu colonization started the fifth day, showing not only less colonization but also a great inhibitory effect.

It is necessary to mention that during the growth in the laboratory it was found that the corn starch lost consistency the fourth day because of the drying of the starch. However, even so, there was microbiological growth in it.

A common practice is to add antifungal to starch pastes, foreseeing these unwanted colonization’s^21^.These compounds can be harmful to the work and toxic to the operator.

Therefore, the non-addition of other fungicidal compounds in starch could be contemplated, if the biocidal capacity of the implicit daidzein in Kudzu was confirmed in subsequent microbiological investigations.

### SEM analysis

SEM analyses were carried out to evaluate the reversibility of the application of the starches, an important point in restoration of artworks^22^. Thanks to this analysis, significant differences were found between starch adhesives.

**Figure 4:**
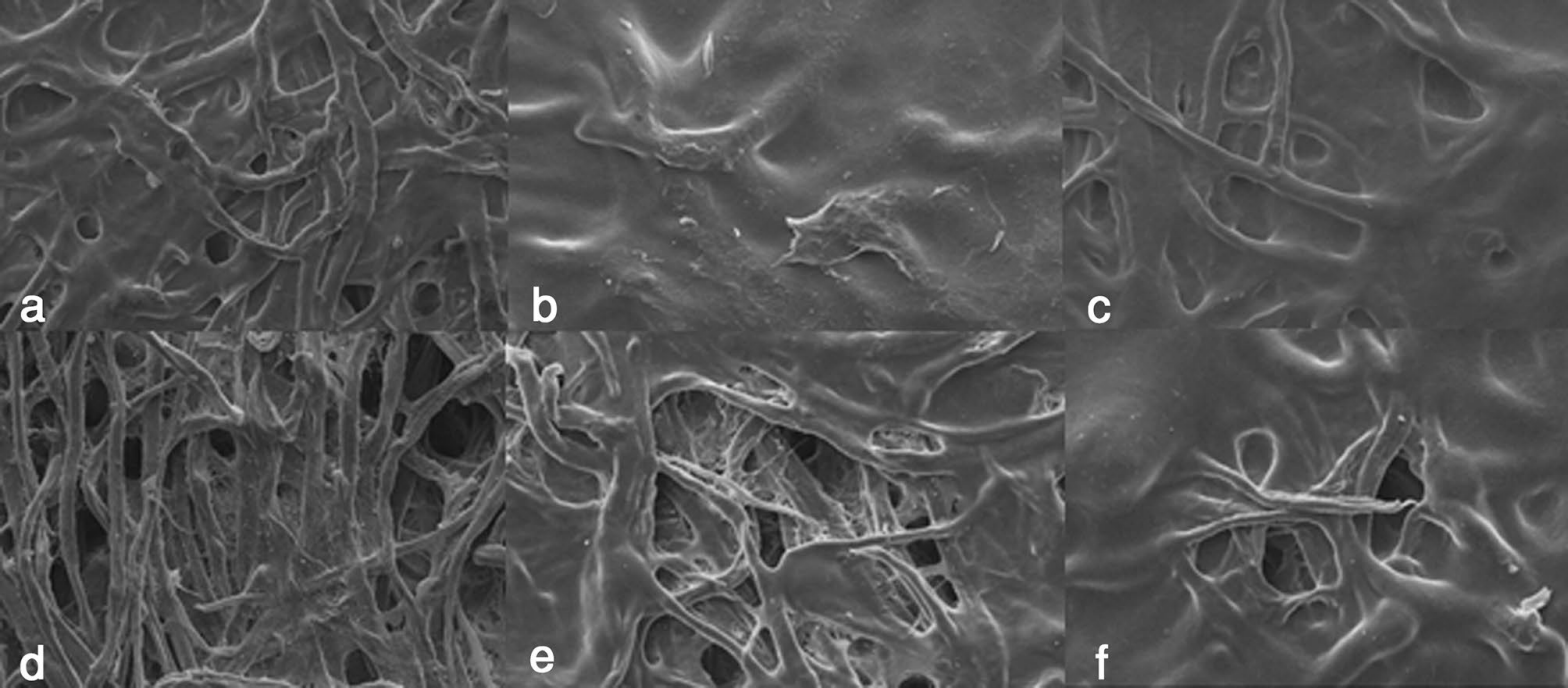
SEM image at 500 × of the samples before removal the starch paste. (**a**) Lineco rice, (**b**) Kudzu, (**c**) Sigma-Aldrich rice, (**d**) Lineco wheat, (**e**) Sigma-Aldrich corn, (**f**) Sigma-Aldrich wheat. Not all starches leaved a uniform layer of adhesive. First, Kudzu followed by Sigma-Aldrich Rice and Lineco rice starch formed a covering layer.

**Figure 5:**
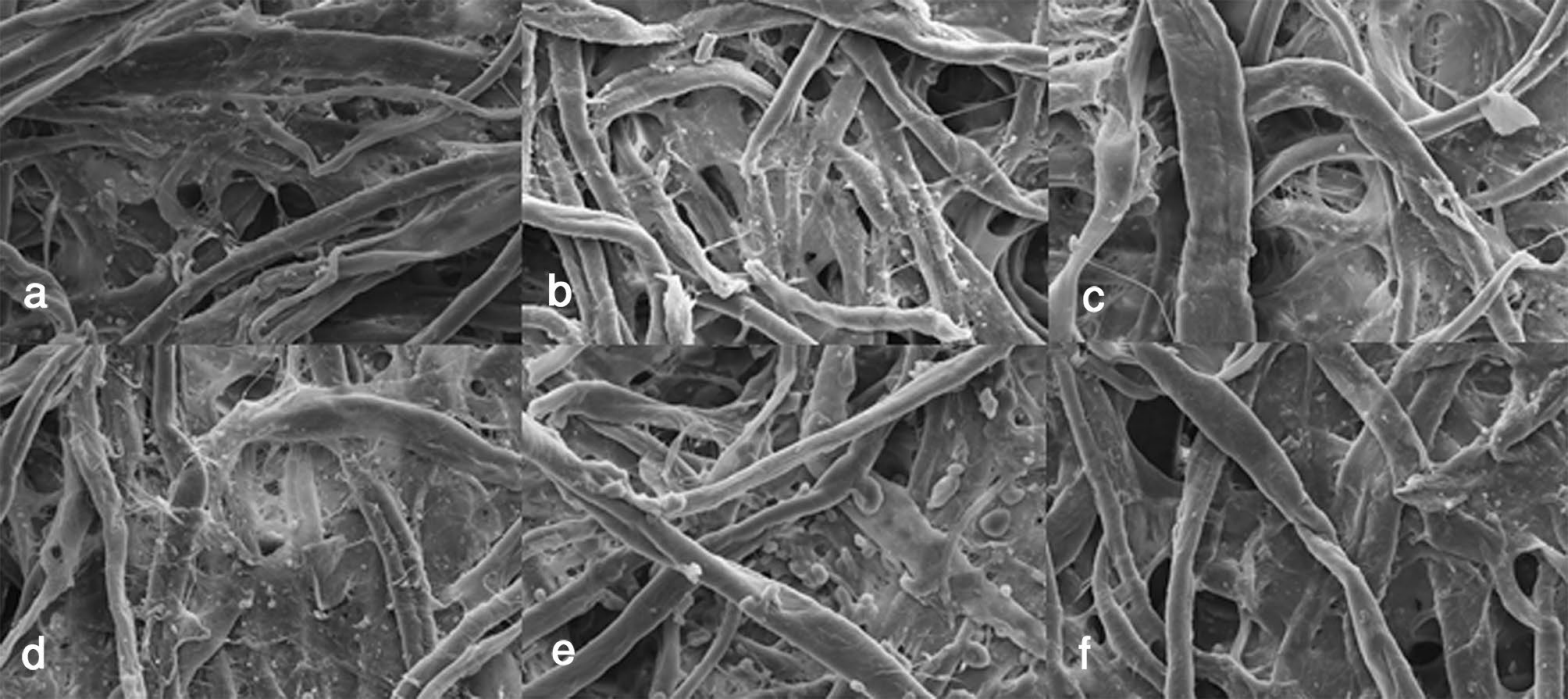
SEM image at 1,000 × of a remove starch paper samples: (**a**) Lineco rice, (**b**) Kudzu, (**c**) Sigma-Aldrich rice, (**d**) Lineco wheat, (**e**) Sigma-Aldrich corn, (**f**) Sigma-Aldrich wheat. Although all the pastes left residues, some of them show small starch granules. It is in Kudzu where the removal of the adhesive at the top of the fibers has been greater.

Regarding the rice starch, it was evenly distributed over the entire surface and even penetrating to inner layers of the paper. Specifically, in the Lineco rice starch, the SEM image before the elimination, determined a paste of adhesive evenly distributed on the surface (Fig. 4a), but with large hollows without adhesive, an issue that indicates a percolation towards the interior of the fibres. Thus, in the surface of the paper the adhesive had been almost completely removed, but there were residues in deeper layers (Fig. 5a). Similarly, Sigma-Aldrich rice starch formed adhesive net with paper fibres creating a uniform base of starch paste (Fig. 4c). After its elimination, there were notable remains on the fibres (Fig. 5c).

Lineco wheat starch applied on paper is placed in lower layers (Fig. 4d), leaving the upper fibres uncovered without adhesive. The elimination of this paste was difficult because of the high penetration of the starch (Fig. 5d). The results obtained from Sigma-Aldrich wheat starch was an irregular coating of the adhesive paste on the surface (Fig. 4f), and a poor elimination from the paper fibres (Fig. 5f). It created compact paste masses that were found at all levels of the paper fibres.

Corn starch generated granules throughout the cellulose layer and a partial coverage of the surface was observed (Fig. 4e). After the removal, leaves granules larger than in the rest of starches, with important remains of adhesive in the lower fibers (Fig. 5e).

Finally, the Kudzu starch created a 100% coverage (Fig. 4b), also had easy removal, only leaving residues on the surface and a minimal penetration in the lower layers of cellulose support (Fig. 5b).

It is necessary to take into account that starch removal procedure had some side effects on the conservation of paper fibres. Indeed, in spite of the fact that starch removal through cotton swabs is considered a non-aggressive method, SEM images proved that several fibres were damaged by friction.

## Conclusions

Kudzu starch is an almost pure compound (close to 100% starch much more than commercialised starches for restoration purposes) that is extracted and processed in a natural and sustainable way providing a high-quality product. This quality is obtained without purification processes, which cheapens the final product. It can be easily purchased in the market and has a similar price to Lineco starches. During the manipulation of the starch, it was observed how Kudzu starch can be able to adapt to different uses or supports and is stable in the time, maintaining its uniformity and reducing its volume slightly. Moreover, although all the test was made in the same concentrations of starches, it could be possible to observe how Kudzu paste could create rigid gel with a great consistency and using lower concentration of starch.

Focusing in the analytical results, all of them pointed out Kudzu starch as a better alternative to the most common used starches. Compared to other starches, Kudzu reported the best results in terms of colour stability, being less affected by the UV radiation. Kudzu also provides a good symbiosis between adhesive and support presenting a good adhesive capacity. Although its adhesion power is not as strong as the one provided by Lineco rice (94%), Kudzu starch can be used for other supports or materials that require different adhesion capacity or are more fragile. Therefore, ensured the best performances in terms of reversibility in comparison with the other studied starches. For that reason, Kudzu is a better option also in these aspects. Finally, the antimicrobial component (daidzein) included in Kudzu starch seemed to be a very beneficial compound to prevent or minimize microorganism’s colonization on several supports.

In view of all these analytical results, the conclusions from this kind of preliminary study suggests that Kudzu starch could be employed in the field of paper conservation and in the protection of Cultural Heritage in general as cleaner, resistance to biological colonization or/and consolidant for different supports.

However, further studies need to be carried out in the future to analyse more in depth all its properties and potential applications, like the microbiological field.

## Methods

### Materials

The most usual starches commercialized within the field of paper restoration have been selected to carry out comparison analysis. Rice and wheat starches were selected from Lineco (Holyoke, Massachusetts), one of the major companies in the production and distribution of conservation products.

In order to compare with high purified starches as standard of the maximum level of quality for its characterization, also starches (from Sigma-Aldrich, USA) with laboratory quality were selected. In this sense, rice and wheat have been selected to the comparison. In this sense, Corn starch although it is the least used, has been considered important to include in this study.

The Kudzu starch powder was supplied by the company Mitoku Company from Japan. This company certifies that their organic product is obtained by extracting the root of Kudzu manually and ecologically, a process that lasts approximately 3 months^23^.

**Figure 6:**
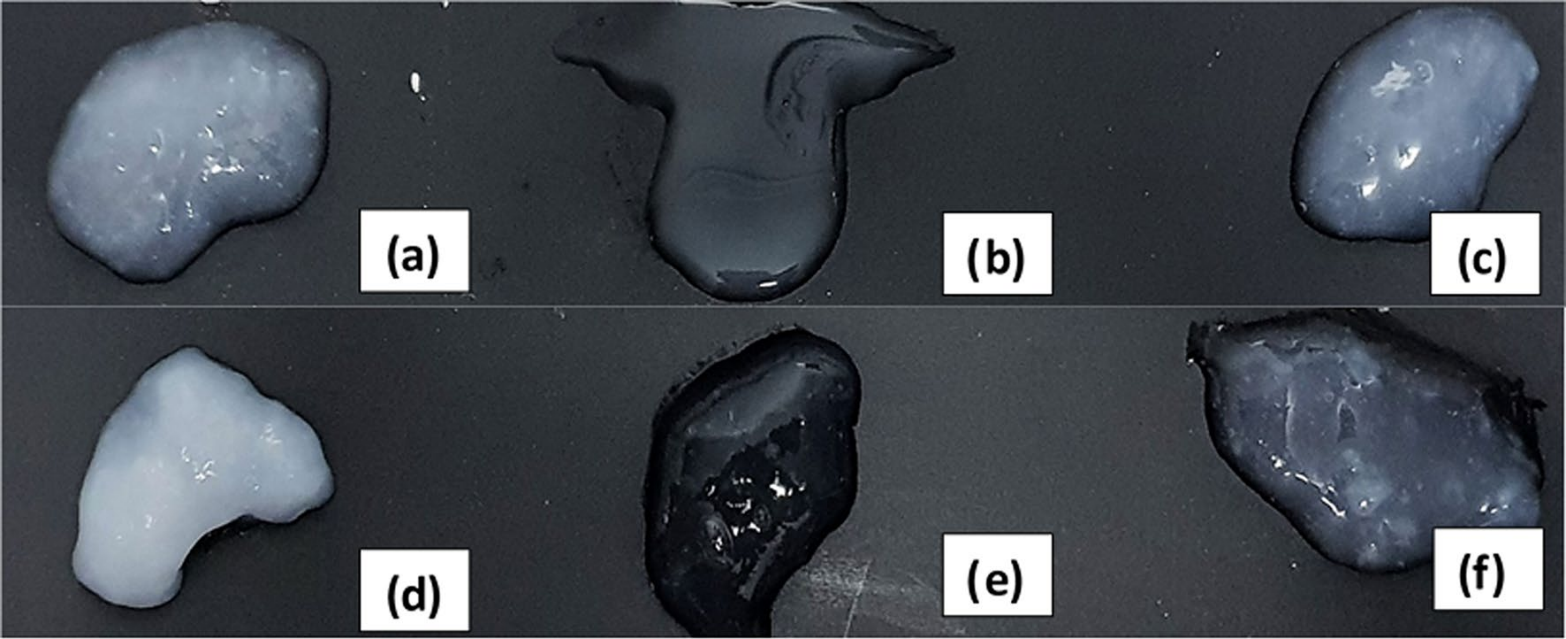
Images of the (**a**). Sigma-Aldrich rice starch (**b**) Sigma-Aldrich corn starch (**c**) Sigma-Aldrich wheat starch (**d**) Lineco wheat starch (**e**) Kudzu starch from Mitoku Company (**f**) Lineco rice starch. Kudzu appearance is noticeably more transparent than usual starches except corn starch that at the same concentration as the others, is much less dense than the rest and that is why it is transparent.

Taking all of these into account, the Kudzu starch was compared with 2–3 starches and two qualities (restoration quality from Lineco and high quality from Sigma-Aldrich): Lineco rice, Lineco wheat, Sigma-Aldrich rice, Sigma-Aldrich wheat and Sigma-Aldrich corn. For this study, all the starches pastes were prepared at the same concentration and with the same cooking methodology giving rise to different pastes (Fig. 6).

The paste was made with 20 ml of cold MiliQ ultrapure water mixed with 4 g of powdered starch, stirring this until the starch dissolved. Each mixture was placed for 30 s in the microwave at 900 W, the mixture was stirred and repeated again 30 s at 900W^6,24^.

### Aging process

The accelerated aging process allows to analyse the chromatic and adhesion changes of the two most common starches: wheat and rice, comparing them with Kudzu starch. For this purpose, a Solarbox HR 1500e camera (from Co.Fo.Me.Gra, Milan, Italy) was used with specific parameters such as: ultraviolet (UV) filter (window glass) at 310 nm, a relative humidity (RH) of 62%, irradiance of 50 W/m^2^, air temperature of 27 °C and a Black Standard Thermometer (BST) of 55 °C.

The test was carried out according to the UNE-EN ISO 11341:2005 norm that systematizes artificial weathering and exposure to artificial radiation of paints and varnishes. It was decided the use this norm because of the lack of a specific regulation for these specific materials.

**Figure 7:**
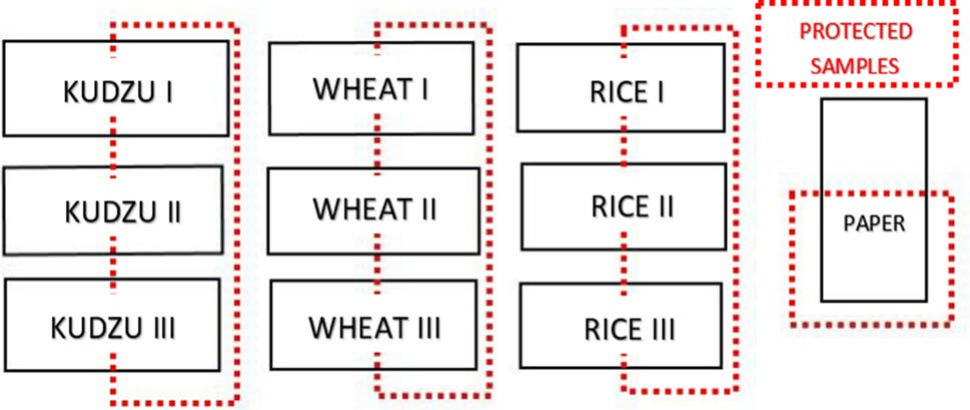
Arrangement of the paper samples with the three starch adhesives applied. Three replicated were carried out for each starch and it was covered the half of the paper to evaluate the UV effect. One control paper was introduced free of starch.

For the preparation of starch paste, 4 g of starch powder was mixed with 20 mL of ultra-pure cold water until dissolved completely, heating the mixture in the microwave^6,24^. The paper used in the accelerated aging process was Fabriano Disegno (from Fabriano, Italy) 4 paper, smooth texture sheet with 220 g produced with 100% cellulose Elemental Chlorine Free (E.C.F.). Three layers of adhesive were applied to each of the samples by brush, once the paste temperature had decreased. After applying the starches, the samples were naturally dried for 24 h and then introduced in the aging chamber. The evaluate the chromatic behaviour of each starch, experiments were performed in triplicate. To consider the UV effect, half of each sample was covered (Fig. 7). Finally, a control paper free of starch was introduced in the test. It is necessary to clarify that for this test only the Lineco starches and Kudzu starches were evaluated because of the limitation in the chamber size.

The samples were periodically measured with the spectrophotometer GretaMacbeth ColorEye XTH (Xrite, Michigan, USA) every 48-h and up to 350 h, obtaining the parameters (L, a and b) within the space (CIELab). The measurements of the samples were determined due to the spectrophotometer spot, not being smaller than 2.5 cm × 5 cm.

### Elemental and molecular analyses during the preparation process

The starch powder was pressed to ensure homogeneity in the results of the chemical analyses. For this process identical pellets of each starch were made using 0.2 g of powder starch that were introduced in a press and were applied 7.0 bar of pressure during 15 s.

The M4 Tornado energy dispersive x-ray fluorescence spectrometer (Bruker Nano GmbH, Berlin, Germany) was used for the elemental analysis. It is equipped with a micro-focus side window Rh X-ray tube powered by a low-power HV generator and cooled by air. It was used a voltage of 40 kV, a current of 600 μA and 100 s of live time for the spectral acquisitions. The vacuum was achieved with a diaphragm pump MV 10 N vario-b. The final focus was carried out by a video-microscope with high magnification (1 mm^2^ areas). The used software was M4 Tornado (Bruker Nano GmbH, Berlin, Germany).

The molecular characterization of the selected starches was made using an InnoRaman ultramobile Raman spectrometer (B&wtek, Newark, USA). The excitation wavelength was 785 nm (the nominal laser power settings on the surface of the samples were 255 mW) and the dispersed Raman signals were measured by a Peltier cooled CCD detector. The spectrometer was supplied with a probe head connected to a micro camera focusing with magnifications of 20× and 50×. The spectra were collected in the spectral range of 100–3,000 cm^−1^ (non-changeable) with a mean spectral resolution of 3.5 cm^−1^. Data acquisition was carried out by the Bwspec 3.26 software package and the analysis of the results was undertaken by the Omnic 7.2 software (Nicolet). In both analyses, a random grid of five points was carried out in the surface of each pellet in order to obtain representative results.

### Adhesion test: T-peel

To evaluate the adhesive capacity a tensiometer was used to determine the force used in the detachment of sheet with the starches by a T-peel adhesion test^25,26^. This was carried out using the Instron 5,967 (Barcelona, Spain) tensioning equipment with pneumatic clamps and a 50 N load cell. The preparation of the starch paste was made with ultra-pure water and 6% starch concentrations. It was applied on 10 sheets of polyethylene terephthalate performing by the norm ASTM D1876, each one with a 241 mm × 25 mm dimensions, adhered to another sheet by the paste of each starch. In order to guarantee the representativeness of the obtained results 10 replicates of each starch were carried out. The drying of the samples was 48 h until the test and the tests was carried out in the next 24 h. The samples were evaluated between 100 to 300 mm in order to standardize and to avoid non-representative results.

### Analysis of microbiological growth

Microbiological growth by a sedimentation process was performed in order to evaluate a preliminary way and as a first approach to the biocidal or resistance capacity of the daidizein implicit on Kudzu, against the other starches.

The different starches were prepared at 6% with ultra-pure water (using the microwave to facilitate the dissolution). Each starch paste was poured into one sterilized Petri dishes (20 mL in each one) and sealed perfectly before their exposition. In order to select the best environment, the dishes were placed in a library for 24 h to be exposed to the microorganisms that generally colonize paper materials.

Subsequently, the exposed samples were sealed and transported to the laboratory where were cultivated for 10 days with a constant temperature of 26 °C. The colony forming units (CFU) were identified, quantified and measured in a Colony Counter 560 (Suntex).

Samples of each different colony were stained with Lactofenol Bleu solution DC (Panreac) and visualized with a Nikon Eclipse E100 microscope at 1000×.

The settlement and the rest of each of them was different, finding areas with more clumps of paste and areas with less.

### SEM (scanning electron microscopy) analysis

To evaluate the reversibility of starches it is necessary to have a microscopic view of the surface. For this purpose, it was prepared samples with Fabriano Disegno 4 paper, previously described, joining two pieces of paper of 3 × 4 cm for each studied starch, and let them dry 48 h. Then, the pieces were separated, and the remainder starch was removed with a cotton swab with ultra-pure water was used for the starch elimination. The SEM analysis was carried out through a Carl Zeiss EVO 40 (Oberkochen, Germany) that allows to visualize the mesh of the vegetable fiber of the paper, as well as, the full or not removal of the adhesive. The conditions of observation and measurement were: high vacuum, a voltage of 20 kV, and a working distance of 8–11 mm. The samples, previously metalized with gold (15 nm), were mounted on an aluminium sample holder with graphite tape (electrically conductive) and observed and photographed at different magnifications (first 100×, later 500× and finally, 1000×).
